# The combination of oxaliplatin and anti-PD-1 inhibitor promotes immune cells infiltration and enhances anti-tumor effect of PD-1 blockade in bladder cancer

**DOI:** 10.3389/fimmu.2023.1085476

**Published:** 2023-03-07

**Authors:** Zihan Zhao, Siyang Liu, Rui Sun, Wenjie Zhu, Yulin Zhang, Tianyao Liu, Tianhang Li, Ning Jiang, Hongqian Guo, Rong Yang

**Affiliations:** ^1^ Department of Urology, Affiliated Drum Tower Hospital, Medical School of Nanjing University, Institute of Urology, Nanjing University, Nanjing, China; ^2^ Department of Urology, Nanjing Drum Tower Hospital Clinical College of Jiangsu University, Nanjing, China

**Keywords:** bladder cancer, oxaliplatin, immunotherapy, combination therapy, immune checkpoint inhibitors, tumor immune microenvironment

## Abstract

**Introduction:**

Bladder cancer (BLCA) is a highly malignant tumor of the urinary system, but the prognosis and survival rates have little improvement based on current therapeutic strategy. Immune checkpoint inhibitors (ICIs) therapy revolutionized the treatment of BLCA, but the clinical application of ICIs is limited by low response rate. Oxaliplatin (OXP), a second line chemotherapy drug for BLCA, may reshape the tumor immune microenvironment (TIME) *via* recruiting immune cells. Here, we conducted the study of oxaliplatin combined with anti-PD-1 inhibitor in BLCA mice models.

**Methods:**

The 6-8 weeks old female C57BL/6J mice were used to establish subcutaneous model of bladder tumor. After tumors developed, mice were given tail vein injections of PBS or oxaliplatin (2.5 mg/kg) and/or anti-PD-1 antibody (10 mg/kg). Tumor tissue samples and peripheral blood mononuclear cell (PBMC) were collected to systemically evaluate the efficiency and safety of combination OXP and anti-PD-1 inhibitor. The change of immune cells populations and the corresponding phenotypic diversity in TIME and PBMC were analysed by flow cytometry.

**Results:**

Tumor growth experiments clarified that the combination therapy was more efficient than medication alone. Flow cytometry analysis of tumor samples showed significant differences between untreated and treated mice. Oxaliplatin influences the TIME by increasing immune cells infiltration, including CD3^+^ T cells, CD4^+^ T cells, CD8^+^ T cells, dendritic cells (DC cells) and natural killer cells (NK cells). As for infiltrating cells, oxaliplatin upregulated the expression of CD134 and downregulated TIM-3 of CD4^+^ T cells, downregulated the PD-L1 expression of DC cells, which contributed to improve the anti-tumor effect and the treatment response of ICIs. Additionally, the evaluation of PBMC found that there were no significant changes in immune cell subsets and phenotypes, which validated the safety of the combination therapy. These results show the therapeutic potential for the combination of OXP and anti-PD-1 inhibitor in BLCA.

**Conclusion:**

OXP could increase the infiltration of immune cells in TIME to promote the anti-tumor activity of anti-PD-1 inhibitor. The present research provided an appropriate rationale of combination chemotherapy and immunotherapy therapy for BLCA.

## 1 Introduction

Bladder cancer (BLCA) is one of the most common genitourinary cancers, which involves an increasing incidence and mortality (1). Although approximately 75% of patients with BLCA present non-muscular invasive tumors, 50%-70% of patients will relapse within five years and 10%-30% may progress to muscular infiltration stage (2, 3). Cisplatin-based chemotherapy is recommended as the first-line standard therapy for advanced bladder cancer (4), but about 40% patients have no response to the treatment (5) and about 50% patients with muscular invasive bladder cancer (MIBC) are ineligible (6).

In recent years, the immune checkpoint inhibitors (ICIs) represented by anti-PD-1 inhibitor have revolutionized the treatment of BLCA because of the strong immunogenicity in tumor microenvironment (TME) and the high mutational burden of BLCA (7). Although the ICIs therapy achieved a relatively long term tumor remission, only less than 30% BLCA patients benefit from it, which limited the clinical application of ICIs (8) (9). Therefore, there is a great interest in developing combination therapy strategy to enhance efficacy and precision of ICIs therapy, including combinations with chemotherapy, radiation therapy, targeted therapy, adaptive cell therapy, and so on (10).

Chemotherapy-induced cancer cell death is thought to promote tumor antigen release and antigen presentation and stimulate immune effectors, which may enhance the anti-tumor efficacy of immunotherapy (10). Oxaliplatin (OXP), a second-line platinum-based chemotherapeutic drug for BLCA, has been reported presumably act as a booster of the immune system to mediate the cancer-immune interface (11). It not only binds to DNA to interfere with DNA and RNA synthesis like cisplatin, but also induces immunogenic cell death (12), enhanced the function and phenotypical maturation of DCs to stimulate proliferation of T cells in melanoma (13). However, the research about four preclinical tumor models have found that the antitumor activity of combinations of chemotherapy and ICI is model-dependent (14). In MB49 bladder cancer model, the combination of cytotoxic regimens with anti-PD-1 inhibitor or anti-PD-L1 inhibitor produced different consequences. Therefore, it’s necessary to comprehensively assess the anti-tumor activity of the combination regimen of oxaliplatin with anti- PD-1 inhibitor in bladder cancer.

**Figure 1:**
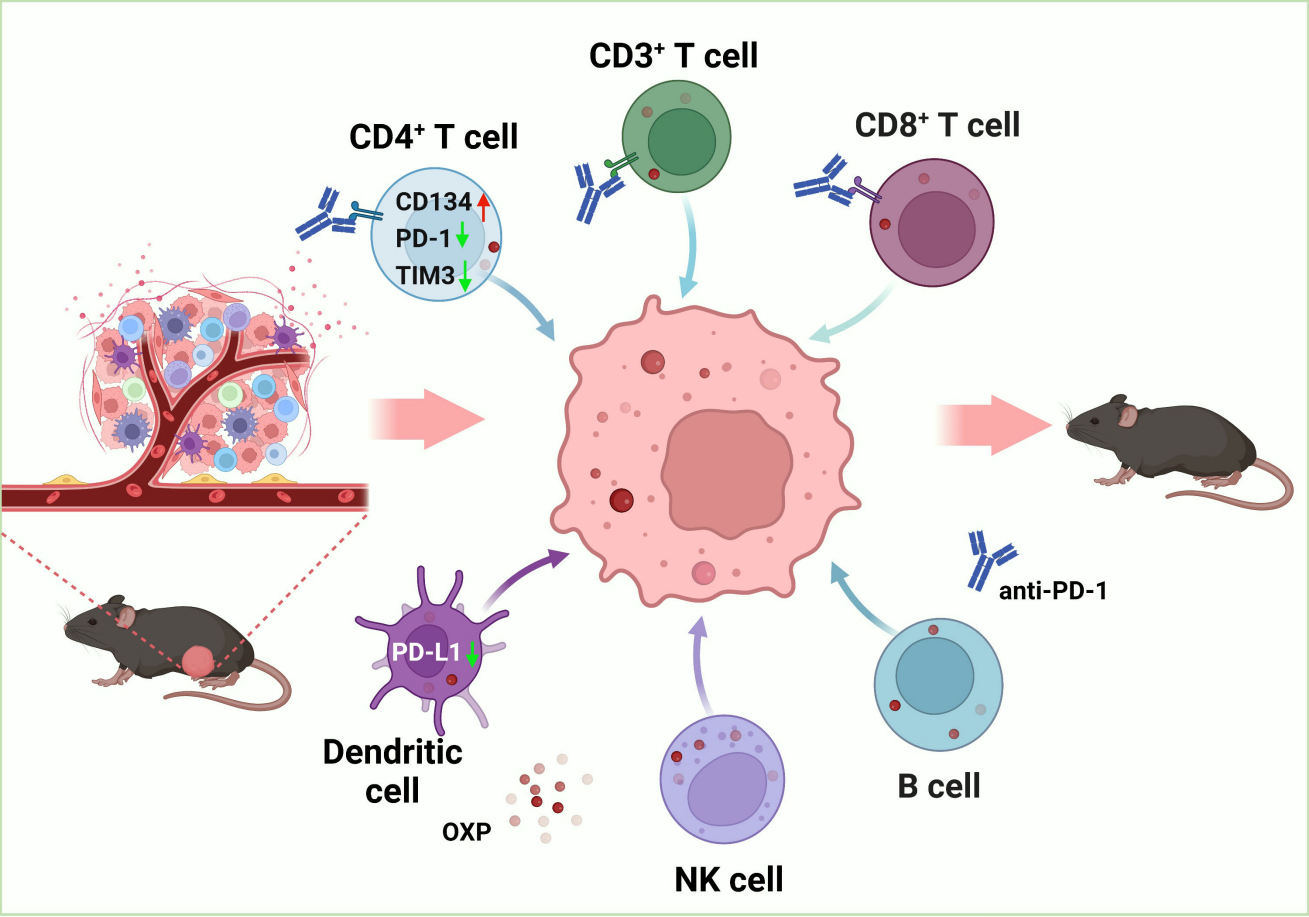
Schematic diagram of the therapeutic mechanism for combination of OXP and anti-PD-1 inhibitor in bladder cancer tumor immune microenvironment.

To explore the potential outcomes of the combination of oxaliplatin with anti- PD-1 inhibitor, we evaluated therapeutic effects of combination treatment and analyzed the consequences of the TIME in bladder cancer. We found that the combination therapy was more efficient in anti-tumor than medication alone. Oxaliplatin alters the TIME by increasing immune cells infiltration, such as CD3^+^ T cells, CD4^+^ T cells, CD8^+^ T cells, dendritic cells (DC cells) and natural killer (NK) cells. As for infiltrating immune cells, oxaliplatin upregulated the expression of CD134 and downregulated TIM-3 of CD4^+^ T cells, downregulated the PD-L1 expression of DC cells, which contributed to improve the anti-tumor effect and the treatment response of ICIs. Besides, there were no significant changes in immune cell subsets and phenotypes in peripheral blood mononuclear cell (PBMC), which validated the safety of the combination therapy. In conclusion, we verified that the combination of OXP and anti-PD-1 inhibitor to promote immune cells infiltration and enhance anti-tumor effect are potential therapeutic strategy for bladder cancer **Figure 1**.

## 2 Materials and methods

### 2.1 Cell culture

The murine bladder cancer cell line MB49 was kindly gifted by Professor Haibo Shen (Shanghai Jiao Tong University, China). The MB49 was cultured in RPMI 1640 (Gibico) supplemented with 10% fetal bovine serum (Gibico) and 1× Penicillin-Streptomycin (Gibico) at 37°C under 5% CO2 in humidified incubator.

### 2.2 Establishment and measure of intradermal mouse tumor model

All experimental animal protocols followed the regulations of the People’s Republic of China on the Administration of Laboratory Animals, and all animal procedures in this work were approved by the Animal Experimental Committee of Nanjing Drum Tower Hospital. 6-8 weeks old female C57BL/6J mice were purchased from GemPharmatech. 1×10^5^ MB49 cells were injected subcutaneously into the right flank of mice to establish a tumor model. The average tumor volume was evaluated every two days. When the average tumor volume of mice reached 80 mm³, mice were randomized and the first treatment was administered. All mice were randomly divided into four groups (5 mice per group) for distinct treatments: (I) PBS group (treated with PBS), (II) OXP group (treated with Oxaliplatin), (III) anti-PD-1 group (treated with anti-PD-1 inhibitor), and (IV) OXP + anti-PD-1 group (treated with anti-PD-1 inhibitor after treated with OXP 2 days).

### 2.3 Treatment strategy

**Figure 2:**
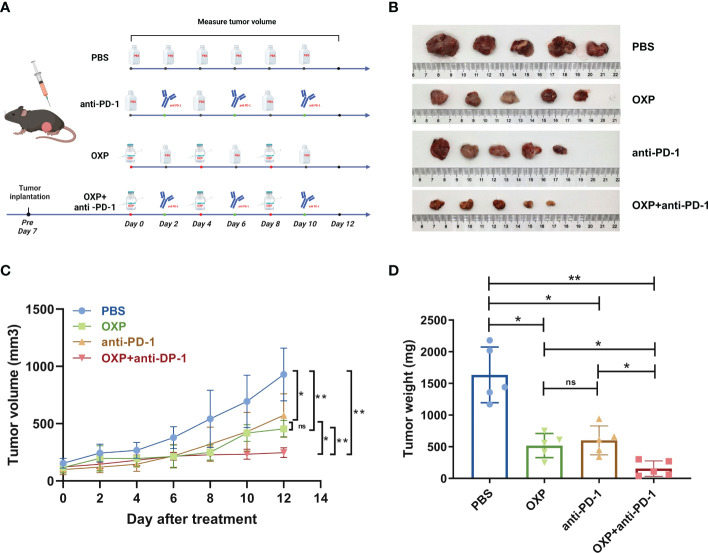
*In vivo* anti-tumor effect of combination OXP and anti-PD-1 inhibitor in C57BL/6 mice models bearing MB49 tumor (n = 5). **(A)** Experimental design and treatment process. **(B)** Excised tumor images. **(C)** Tumor growth curve. **(D)** Tumor weights of excised tumors. *P < 0.05; **P < 0.01; ns P > 0.05.

The treatment timeline was shown in **Figure 2A**. Briefly, PBS-treated tumor-bearing mice were used as control. For OXP group, oxaliplatin (purchased from Shandong Boyuan Pharmaceutical Co. Ltd.) was administered by tail vein injection every 4 days at a dose of 2.5 mg/kg. For anti-PD-1 group, anti-PD-1 (Bioxcell, RMP1-14, Car#BE0146) was administered by intraperitoneal injection every 4 days at a dose of 10 mg/kg. For OXP + anti-PD-1 group, administration was performed in the same manner as above. 12 days after the first administration of treatment, all mice were sacrificed and tumor tissues were surgically removed. Before sacrifice, blood was collected through orbital puncture in EDTA tubes and extracted the PBMC, immediately.

### 2.4 Flow cytometry

All tumor tissue samples and PBMC were collected for analyzing the TIME and related changes by flow cytometry. Cells were firstly suspended in FACS buffer (PBS, supplemented with 2% FBS) and incubated with TruStain FcX™ PLUS (anti-mouse CD16/32, 1:100 # 156604) for 5 minutes. Next, 100µL diluted antibodies solution was used to suspend cells and incubated at 4°C for 30 minutes in the dark. After washing twice with FACS buffer, the resuspended cells were prepared for analyzed, the gating strategies were shown in **Figures S1**-**S3**. The three flow cytometry staining panels designed to analyze cell components and cell surface markers were shown in **Table 1**. All antibodies were purchased from BioLegend, Inc.

**Table 1:** Antibodies information for flow cytometry.

Marker	Conjugate/Tag	Cat#
Panel 1		
anti-mouse CD45	AF-700	103128
anti-mouse CD3	PE	124609
anti-mouse CD4	APC	100516
anti-mouse CD8a	PB	100725
anti-mouse CD19	FITC	152404
anti-mouse NK1.1	PE/Cy5	108714
Zombie Aqua Fixable Viability Kit	AmCyan	423102
Panel 2		
anti-mouse CD3	PE	124609
anti-mouse CD4	APC	100516
anti-mouse CD8a	PB	100725
anti-mouse CD39	PE/Cy7	143806
anti-mouse CD69	PE/Cy5	104510
anti-mouse CD103	BV 785	121439
anti-mouse CD134	BV 711	119421
anti-mouse PD-1	FITC	135214
anti-mouse Tim 3, CD366	PerCP/Cy5.5	119718
Zombie Aqua Fixable Viability Kit	AmCyan	423102
Panel 3		
anti-mouse CD45	AF-700	103128
anti-mouse CD11c	APC	117310
anti-mouse CD40	PB	124626
anti-mouse CD80	PE	104708
anti-mouse CD86	FITC	105005
anti-mouse PD-L1, CD274, B7-H1	BV 605	124321
Zombie Aqua Fixable Viability Kit	AmCyan	423102

### 2.5 Statistical analysis

For the analysis of experimental data, statistical analyses were performed using GraphPad Prism software (version 8.0). Statistical significance over groups were analyzed using one-way ANOVA. Data are presented as the mean ± SD in all figures with error bars. In all figures, ns, P > 0.05; *P < 0.05; **P < 0.01; ***P < 0.001; ****P < 0.0001.

## 3 Results

### 3.1 Combination of OXP and anti-PD-1 inhibitor enhances the anti-tumor efficacy

To explore the anti-tumor potential of combination therapy strategy, we first established the MB49 syngeneic orthotopic model. We found that the volume of tumor grew more slowly in the drug-treated groups, and the combined group was more effective than other single drug groups (**Figures 2B, C**). Although both OXP group and anti-PD-1 group resulted in smaller tumor volumes compared to the PBS group, tumor volumes in OXP+anti-PD-1 group were significantly smaller than OXP group and anti-PD1 group (**Figure 2C**, all P < 0.05). Additionally, the combination of OXP and anti-PD-1 inhibitor significantly decreases the tumor weight (**Figure 2D**, P < 0.01). As the experimental results show, the combination therapy strategy has stronger capacity than either regimen used OXP or anti-PD-1 inhibitor alone.

### 3.2 Combination of OXP and anti-PD-1 inhibitor promotes immune cells infiltration

**Figure 3:**
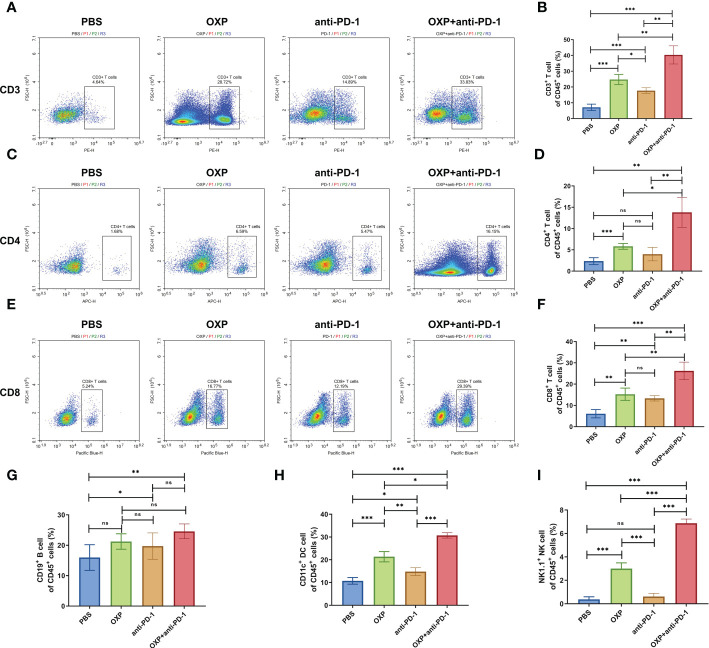
Composition of major immune cells populations in tumor immune microenvironment. **(A)** Representative flow cytometric profiles of CD3^+^ T cells. **(B)** The percentage of CD3^+^ T cells of CD45^+^ cells. **(C)** Representative flow cytometric profiles of CD4^+^ T cells. **(D)** The percentage of CD4^+^ T cells of CD45^+^ cells. **(E)** Representative flow cytometric profiles of CD8^+^ T cells. **(F)** The percentage of CD8+ T cells of CD45^+^ cells. **(G)** The percentage of CD19^+^ B cells of CD45^+^ cells. **(H)** The percentage of CD11c^+^ DC cells of CD45^+^ cells. **(I)** The percentage of NK1.1^+^ NK cells of CD45^+^ cells. ns P > 0.05, *P < 0.05; **P < 0.01; ***P < 0.001.

To evaluate potential synergistic mechanism of OXP with anti-PD-1 inhibitor, we characterized the atlas of TIME in different groups by flow cytometry. Firstly, we systemically analyzed the major immune cell populations of TIME, including CD3^+^ T cells, CD4^+^ T cells, CD8^+^ T cells, B cells, DC cells and NK cells and found that the total immune infiltrate profiles in each group showed a strong heterogeneity (**Figure 3** **)**. We observed that OXP significantly increased the infiltration of CD3, CD4, CD8, CD19, CD11c and NK1.1 compared with PBS treatment (**Figure 3**, all P < 0.01), while anti-PD-1 inhibitor only promoted the infiltration of CD3^+^ T cells, CD8^+^ T cells, CD19^+^ B cells, and CD11c^+^ DC cells compared with PBS treatment (**Figure 3**, all P < 0.05). Moreover, OXP was more efficient to recruiting immune cells versus anti-PD-1 inhibitor, OXP group significantly improved the infiltration of CD3^+^ T cells, CD4^+^ T cells, CD11c^+^ DC cells and NK1.1^+^ NK cells compared with anti-PD-1 group (**Figure 3**, all P < 0.05). Most importantly, because of the synergistic effect of OXP and anti-PD-1, the combination therapy group significantly increased the infiltration of CD3^+^ T cells, CD4^+^ T cells, CD8^+^ T cells, DC cells and NK cells compared with the monotherapy group (**Figure 3**, all P < 0.05).

### 3.3 Combination of OXP and anti-PD-1 inhibitor reshapes the phenotype of immune cells

**Figure 4:**
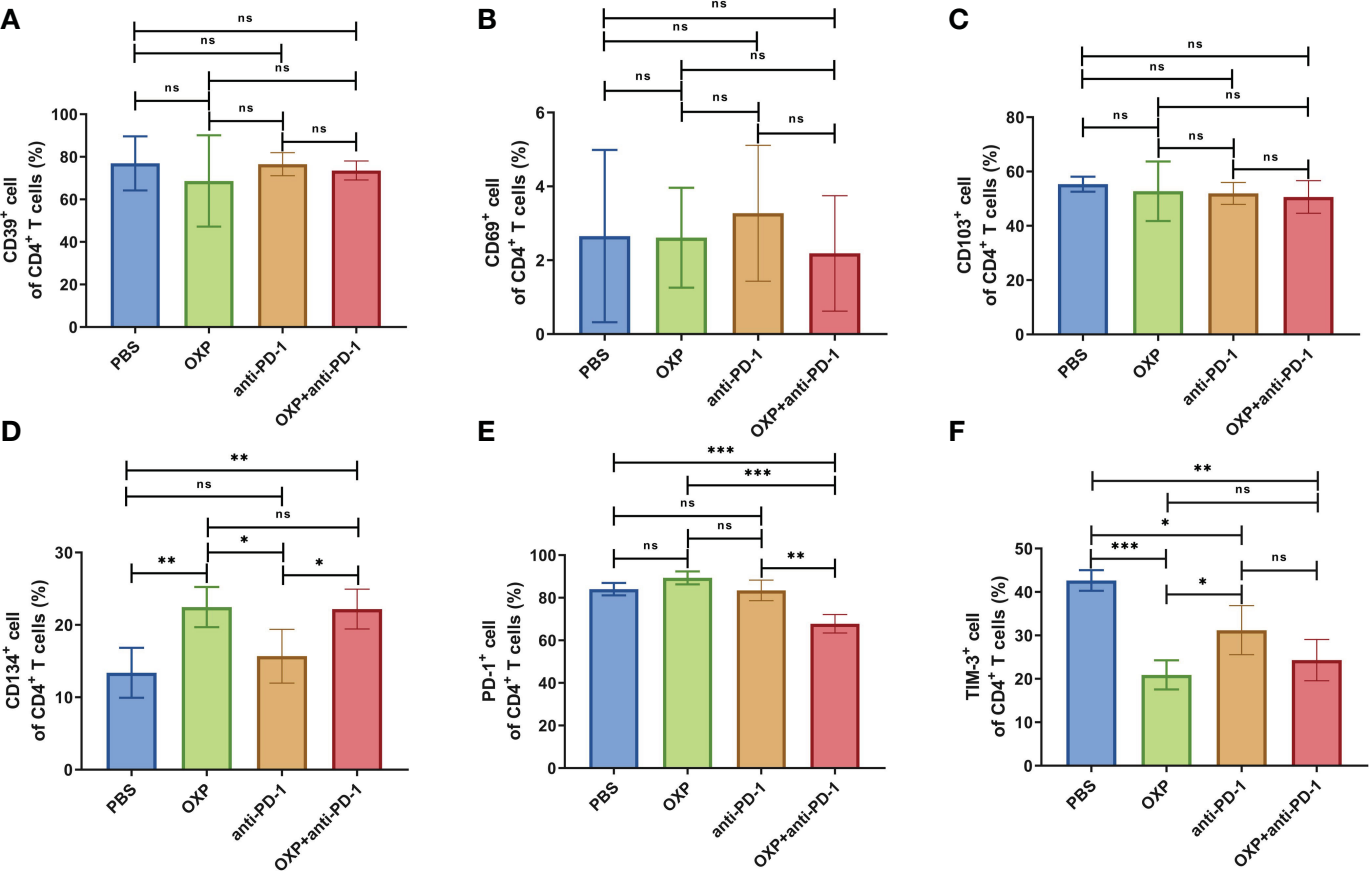
The phenotype of CD4^+^ T cells in TIME. **(A)** The percentage of CD39^+^ cells of CD4^+^ T cells. **(B)** The percentage of CD69^+^ cells of CD4^+^ T cells. **(C)** The percentage of CD103^+^ cells of CD4^+^ T cells. **(D)** The percentage of CD134^+^ cells of CD4^+^ T cells. **(E)** The percentage of PD-1^+^ cells of CD4^+^ T cells. **(F)** The percentage of TIM-3^+^ cells of CD4^+^ T cells. ns P > 0.05, *P < 0.05; **P < 0.01; ***P < 0.001.

**Figure 5:**
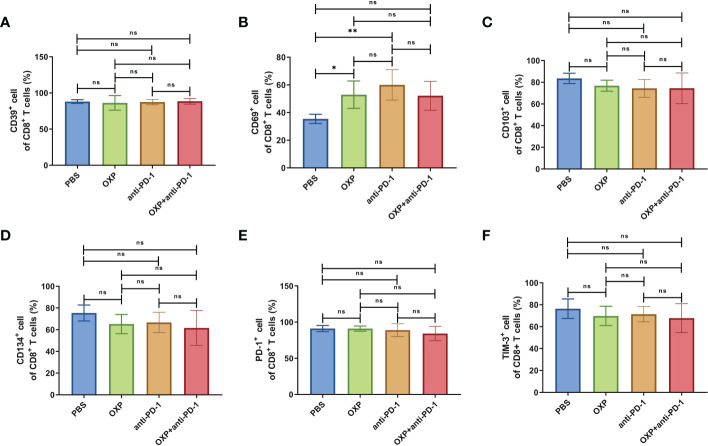
The phenotype of CD8^+^ T cells in TIME. **(A)** The percentage of CD39^+^ cells of CD8^+^ T cells. **(B)** The percentage of CD69^+^ cells of CD8^+^ T cells. **(C)** The percentage of CD103^+^ cells of CD8^+^ T cells. **(D)** The percentage of CD134^+^ cells of CD8^+^ T cells. **(E)** The percentage of PD-1^+^ cells of CD8^+^ T cells. **(F)** The percentage of TIM-3^+^ cells of CD8^+^ T cells. ns P > 0.05, *P < 0.05; **P < 0.01.

**Figure 6:**
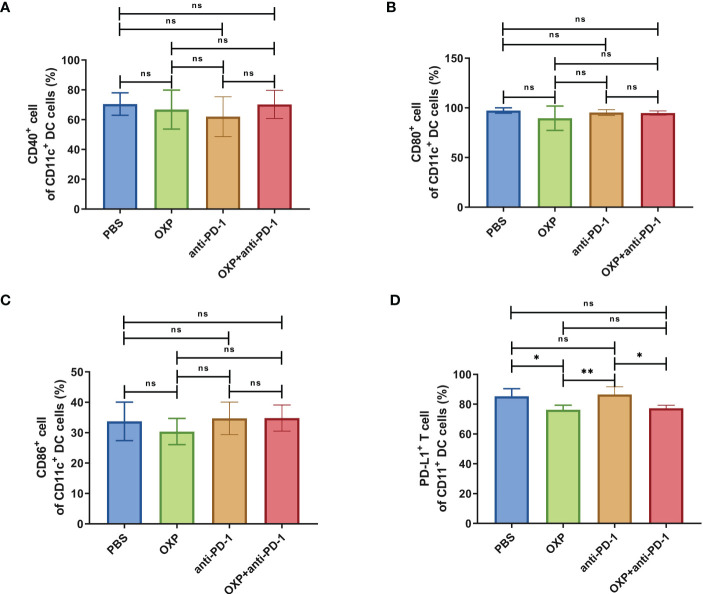
The phenotype of CD11c^+^ DC cells in TIME. **(A)** The percentage of CD40^+^ cells of CD11c^+^ DC cells. **(B)** The percentage of CD80^+^ cells of CD11c^+^ DC cells. **(C)** The percentage of CD86^+^ cells of CD11c^+^ DC cells. **(D)** The percentage of PD-L1^+^ cells of CD11c^+^ DC cells. ns P > 0.05, *P < 0.05; **P < 0.01.

Next, according to the above results, we profoundly investigated the potential phenotype alterations of T cells and DC cells of each group., including CD39, CD69, CD103, CD134, PD-1, TIM-3, CD40, CD80, CD86, and PD-L1. In CD4^+^ T cells population, there were no significant differences of CD39, CD69, and CD103 expression in all groups (**Figures 4A–C**, all P > 0.05). Interestingly, the expression of CD134 was significantly upregulated in OXP group and combination therapy group compared with PBS group and anti-PD-1 group (**Figure 4D**, all P < 0.05), but there was no significant difference between anti-PD-1 group and PBS group. The expression of PD-1 in CD4^+^ T cells decreased after the combined treatment (**Figure 4E**, P < 0.01), but had little change in mice exposed to oxaliplatin or anti-PD-1 alone (**Figure 4E**, P > 0.05). As for TIM-3, the expression of TIM-3 was decreased after treatment with OXP (**Figure 4F**, P < 0.001) or anti-PD-1 inhibitor (**Figure 4F**, P < 0.05) alone compared to the PBS group, but TIM-3 expression was upregulated after treatment with anti-PD-1 inhibitor alone compared to treatment with OXP alone (**Figure 4F**, P < 0.05). Similarly, we also analyzed the phenotypes changes of CD8^+^ T cells, discovered that the expression of CD39, CD103, CD134, PD-1, and TIM-3 had no obvious difference (**Figure 5**, all P> 0.05). Although both OXP and anti-PD-1 inhibitor increased the expression of CD69 in CD8^+^ T cells, but the fraction of CD69^+^ CD8^+^ T cells in combination therapy group was no evident difference compared with the PBS group (**Figure 5B**, P > 0.05). As for DC cells, while the expression of CD40, CD80 and CD86 had no distinct differences in all groups (**Figures 6A–C**, P > 0.05), significantly decreased expression of PD-L1 was observed in OXP-treated group (**Figure 6D**, P < 0.05).

### 3.4 Combination therapy strategy is safe and feasible with little effect on PBMC

**Figure 7:**
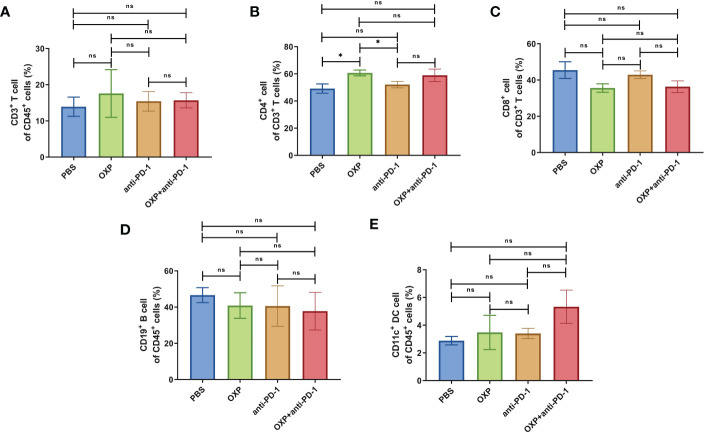
The composition of major immune cells populations in PBMC. (n = 3). **(A)** The percentage of CD3^+^ T cells of CD45^+^ cells. **(B)** The percentage of CD4^+^ T cells of CD3^+^ T cells. **(C)** The percentage of CD8^+^ T cells of CD3^+^ T cells. **(D)** The percentage of CD19^+^ B cells of CD45^+^ cells. **(E)** The percentage of CD11c^+^ DC cells of CD45^+^ cells. ns P > 0.05, *P < 0.05.

**Figure 8:**
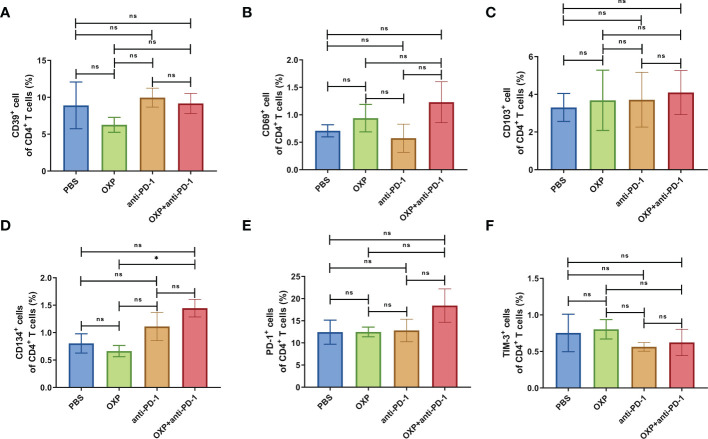
The phenotype of CD4^+^ T cells in PBMC. **(A)** The percentage of CD39^+^ cells of CD4^+^ T cells. **(B)** The percentage of CD69^+^ cells of CD4^+^ T cells. **(C)** The percentage of CD103^+^ cells of CD4^+^ T cells. **(D)** The percentage of CD134^+^ cells of CD4^+^ T cells. **(E)** The percentage of PD-1^+^ cells of CD4^+^ T cells. **(F)** The percentage of TIM-3^+^ cells of CD4^+^ T cells. ns P > 0.05, *P < 0.05.

**Figure 9:**
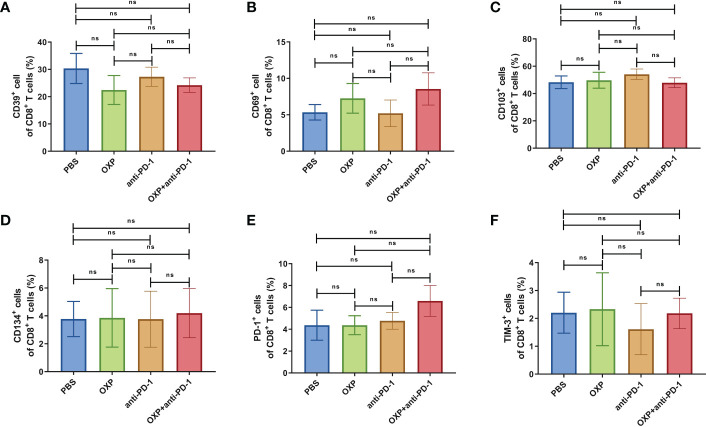
The phenotype of CD8^+^ T cells in PBMC. **(A)** The percentage of CD39^+^ cells of CD8^+^ T cells. **(B)** The percentage of CD69^+^ cells of CD8^+^ T cells. **(C)** The percentage of CD103^+^ cells of CD8^+^ T cells. **(D)** The percentage of CD134^+^ cells of CD8^+^ T cells. **(E)** The percentage of PD-1^+^ cells of CD8^+^ T cells. **(F)** The percentage of TIM-3^+^ cells of CD8^+^ T cells. ns P > 0.05.

**Figure 10:**
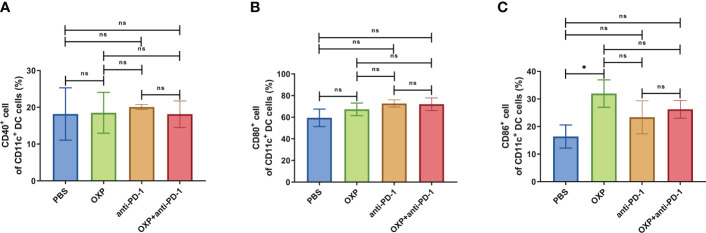
The phenotype of CD11c^+^ DC cells in PBMC. **(A)** The percentage of CD40^+^ cells of CD11c^+^ DC cells. **(B)** The percentage of CD80^+^ cells of CD11c^+^ DC cells. **(C)** The percentage of CD86^+^ cells of CD11c^+^ DC cells. ns P > 0.05, *P < 0.05.

To clarify the safety of the combination of OXP and anti-PD-1 inhibitor, we also investigated the immune cell populations and cell surface markers of PBMC. We found that the percentages of immune cells had no significant difference in different groups, including CD3^+^ T cells, CD4^+^ T cells, CD8^+^ T cells, B cells, and DC cells (**Figure 7**, all P > 0.05), after treated by OXP and anti-PD-1 inhibitor, the phenotype of immune cells in PBMC was not significant alter compared with other groups (**Figures 8**–**10**, all P > 0.05), which confirmed that the combination therapy strategy is safe and feasible with little effect on PBMC.

## 4 Discussion

Bladder cancer is a highly malignant urological tumor with increasing morbidity and mortality rates and a high recurrence rate (15). From the radical cystectomy to cisplatin-based neoadjuvant chemotherapy and radiotherapy, the treatment paradigm of BLCA has developed a lot but the prognosis and survival rates have little improvement in the past 30 years (16, 17). With the improved understanding of molecular biology and genetics, immunotherapy, represented by ICIs, appears to stand with tremendous potential to treat cancers (18). Pembrolizumab, anti-PD-1 inhibitor, has been approved as a feasible treatment choice for cisplatin-ineligible patients (19) or patients with high risk non-MIBC unresponsive to bacillus Calmette-Guerin (BCG) (9). However, the ICIs monotherapy still faces limitations about low response rate and unpredictable performance (20).

Previous research reported that platinum-based chemotherapy, a conventional treatment, may affect the patient’s immune system (21). Some clinical trials also found that the combination of PD-1 (22)/PD-L1 (23) inhibitor with platinum-based chemotherapy would enhance the efficacy and safety of monotherapy. Although the immunogenicity caused by platinum drugs is responsible for the effectiveness of combination therapy, the effect of platinum drugs on the TIME is still unknown, especially in BLCA.

OXP, a second line chemotherapy drug for BLCA, attracted our attention due to the high frequency of cisplatin-resistance in BLCA. A phase II clinical trial confirmed the treatment regime of anti-PD-1 inhibitor plus chemotherapy of gemcitabine and oxaliplatin for advanced biliary tract cancer has a 50% progression free survival (PFS) within 6 months (24). Additionally, Srivastava, et al. reported that OXP could increase recruitment of CAT-T cells to lung tumors, improved tumor response to anti-PD-L1 inhibitor, and enhanced anti-tumor activity (25). OXP is also able to induce tumor-associated macrophages 2 (TAM2) depletion (26) and myeloid-derived suppressor cells (MDSC) decreasing (27), consequently activated CD8+ T cells in tumor-bearing mice. Previous study demonstrated OXP has strong immunogenic momentum and its potential efficiency of combination with ICIs advances for further study. Therefore, we performed this study to evaluate the combination of OXP and anti-PD-1 inhibitor in BLCA mouse models and analyzed the consequences of TIME.

We found that the combination therapy was more efficient in anti-tumor than monotherapy. OXP significantly increased immune cells infiltration and alters the cell surface markers of infiltrating CD4^+^ T cells and DC cells, which contributed to improving the anti-tumor effect and the treatment response of ICIs. Unlike previous discoveries, we found that OXP dominantly influenced CD4^+^ T cells, not CD8^+^ T cells. It is traditionally believed that CD8^+^ T cells are mainly involved in anti-tumor immunity, but researchers have come to realize the importance of CD4^+^ T cells. CD4^+^ T cells can act as recruiters and stimulators of anti-tumor response in cancer immunotherapy (28). Intravesical BCG infusion therapy, the primary treatment for NMIBC, may induce CD4^+^ T cell expansion to anti-tumor (29). A recent study revealed that CD4^+^ T cells also could kill autologous tumor cells directly as cytotoxic immune cells in BLCA (30). Our results demonstrated the CD4^+^ T cells play an essential role in combination OXP and anti-PD-1 therapy, which will further improve the understanding of CD4^+^ T cells population in bladder cancer.

Not only that, but we also found that the expression of CD134 was significantly increased after combination therapy. CD134 (also known as OX40) is a co-stimulatory receptor that is predominantly expressed on activated T cells to regulate the division and survival of conventional T cells (31). Activating CD134 changes tumor immune activation and promotes the efficiency of ICIs (32, 33). The anti-CD134 agonist antibodies increased CD4^+^ T cells in pancreatic cancer mouse models and have a synergistic effect with anti-PD-1 inhibitor to eradicating tumor (34). Additionally, TIM-3 was observed to be significantly decreased in the OXP group, but slightly increased in the combination therapy group compared to the OXP group. TIM-3 is identified as a co-inhibitory receptor which expressed on exhausted T cells. TIM-3 would be recruited to the immunological synapse to inhibit activation of T cells (35). The upregulation of TIM-3 is associated with the dysfunction of T cells and decreasing it would recovery tumor immune response (36). Several combination treatment regimens of anti-PD-1 inhibitor with anti-TIM-3 antibody have completed the phase I/IB clinical trial and exhibited acceptable safety profile (37, 38). Therefore, we speculated that OXP may decrease the expression of TIM-3 in CD4^+^ T cells, but the anti-PD-1 inhibitors may lead to the upregulation of TIM-3, which ultimately resulted in the changes of TIM-3 of CD4^+^ T cells in the combination therapy group.

In addition to T cell populations, we also observed that NK cells and DC cells were significant infiltration in TIME. NK cells are critical members of innate immune and can kill multiple adjacent cells in major histocompatibility complex (MHC) independent manner. NK cells also release several cytokines to increase the expression of human leukocyte antigen (HLA) of tumor cells and recruit DC cells (39, 40). DC cells are key regulator of adaptive immune which present tumor antigen to activate T cell immunity (41). The vaccination strategies in cancer therapies are based on DC cells (42). It has been found that OXP could cause immunogenic cell death of cancer cells (12, 43). OXP stimulated pre-apoptotic exposure of calreticulin (CRT), the post-apoptotic release of high-mobility group box 1 protein (HMGB1), and the secrete of adenosine triphosphate (ATP)(43, 44), which may promote DC cells maturation and antigen presentation to tumor-specific cytotoxic T lymphocytes (44). Besides, we also found that OXP downregulated the PD-L1 expression of DC cells. PD-L1 is the ligand of PD-1, the binding of PD-1 and PD-L1 suppress T cells to inhibit the anti-tumor response (45). Targeting PD-L1 is another immunotherapy which is widely studied (46). Recent studies reported that PD-L1 expression by DC cells is a key regulator of T-cell immunity in cancer, and blocking the PD-L1 of DC cells could enhance the response of T cell to immunotherapy (47, 48). Moreover, we also evaluated the immune cells of PBMC and found no significant changes in immune cell subsets and phenotypes in PBMC, which indicated that the impact of OXP on immune cells may be specificity of tumor tissue.

There were a number of limitations to our study. We performed the study *via* subcutaneous transplantation models. Orthotopic transplantation models may reflect better about the biological characteristics of human tumors. The selection of drug doses and the cut-off time were empirical judgments based on literature and previous experience, and we do not evaluate the influence of different dose of OXP. We analyzed the combination of OXP and anti-PD-1 inhibitor, but other ICIs were not explored, such as anti-PD-L1 inhibitor. Although we have analyzed a variety of cell components and surface markers, these cells can not represent the full picture of TIME, particularly the immunosuppressive cell subpopulation. Previous study found that OXP induced the depletion of TAMs, resulting in a change of the TAM1/TAM2 ratio, while other immunosuppressie cells, such as T regulator cells (Tregs) and myeloid-derived suppressor cells (MDSC) were not affected by the treatment (26). Unfortunatelly, these immunosuppressive cells were not involved in our study. Therefore, the anti-tumor mechanism of combination therapy remains to be investigated to lay a solid foundation for the further clinical trials.

## 5 Conclusion

In conclusion, we verified that chemotherapy could enhance the efficiency of immunotherapy. In BLCA mice models, the combination of oxaliplatin and anti-PD-1 inhibitor could restrict tumor growth and enhance the infiltration of immune cell populations in TIME, including CD3^+^ T cells, CD4^+^ T cells, CD8^+^ T cells, DC cells, and NK cells. CD134^+^CD4^+^ T cells may play an essential role in improving the anti-tumor effect and the treatment response of ICIs. Our present research provided an appropriate rationale of combination chemotherapy and immunotherapy therapy for BLCA.

## Data availability statement

The raw data supporting the conclusions of this article will be made available by the authors, without undue reservation.

## Author contributions

ZHZ, WJZ, RS developed this idea and designed this research. ZHZ, SYL, and YLZ analysed the data. ZHZ, SYL, THL, NJ, and TYL wrote the draft of the manuscript. HQG and RY obtained copies of studies and revised the writing. All authors contributed to the article and approved the submitted version.
