# Supplementary figures and images for: The combination of oxaliplatin and anti-PD-1 inhibitor promotes immune cells infiltration and enhances anti-tumor effect of PD-1 blockade in bladder cancer
Source: Front Immunol. 2023 Mar 7;14:1085476. doi: 10.3389/fimmu.2023.1085476 (PMC10027707; doi:10.3389/fimmu.2023.1085476)

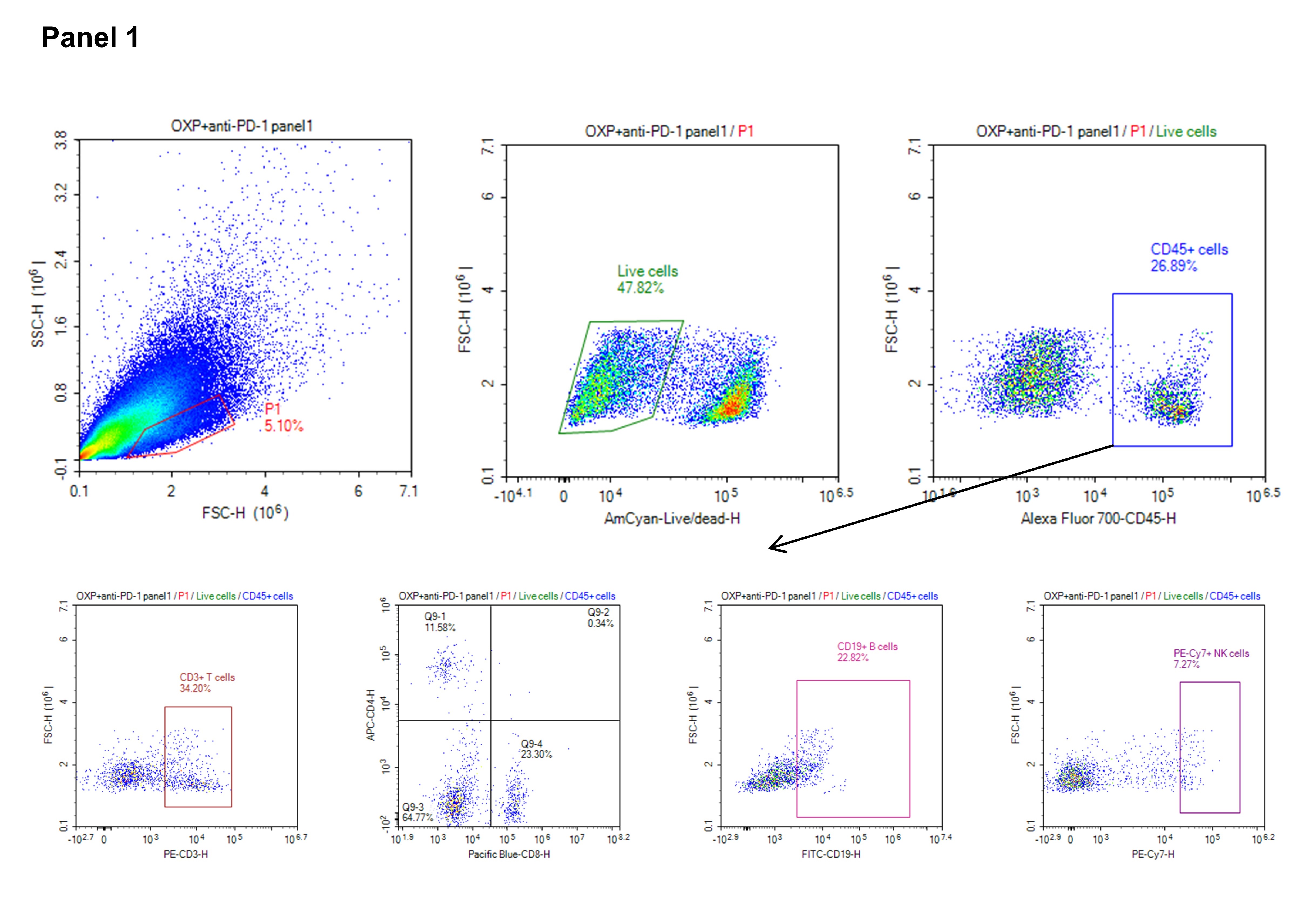

Supplement: Supplementary file 1 [file Image_1.jpeg]

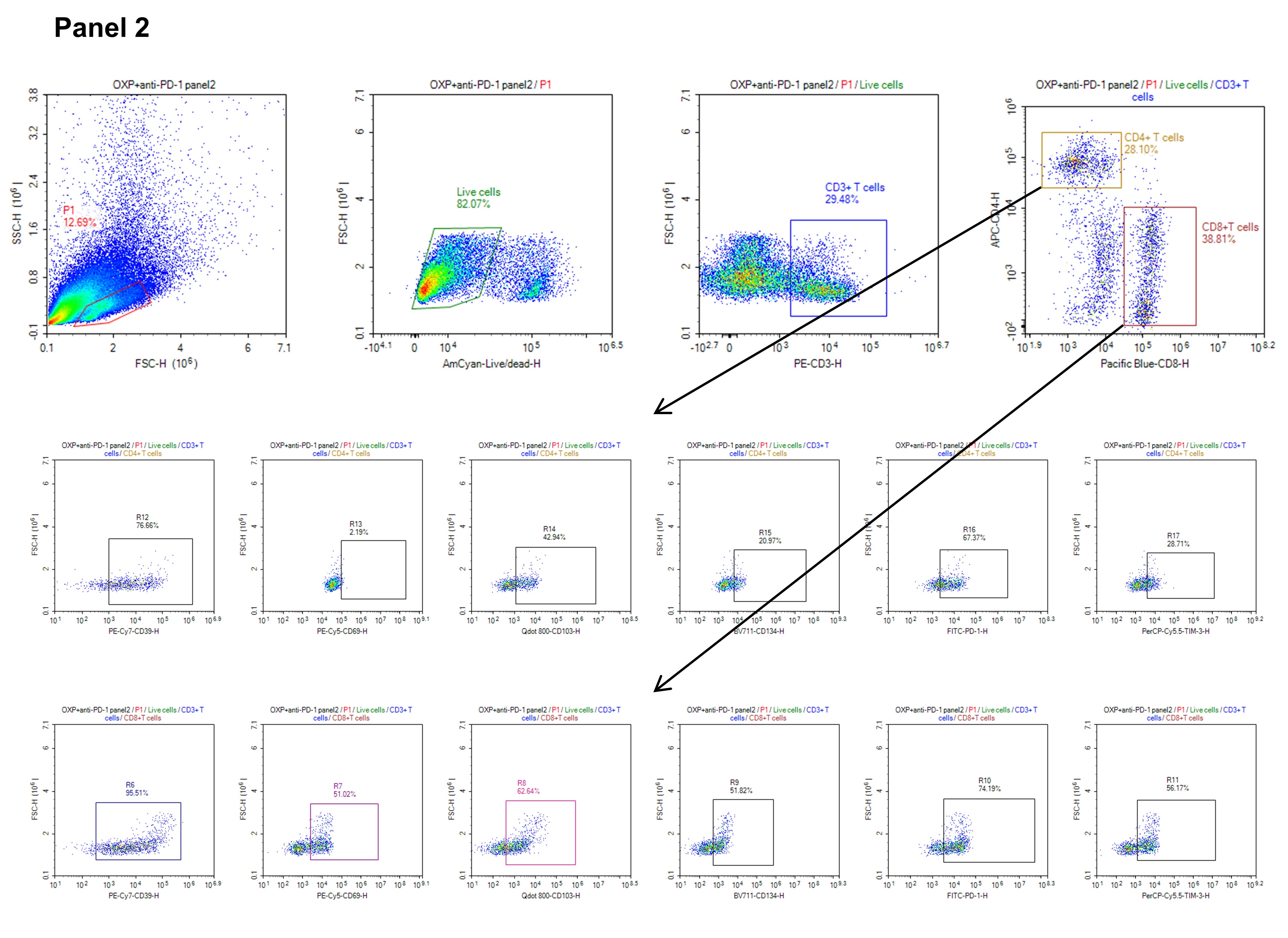

Supplement: Supplementary file 2 [file Image_2.jpeg]

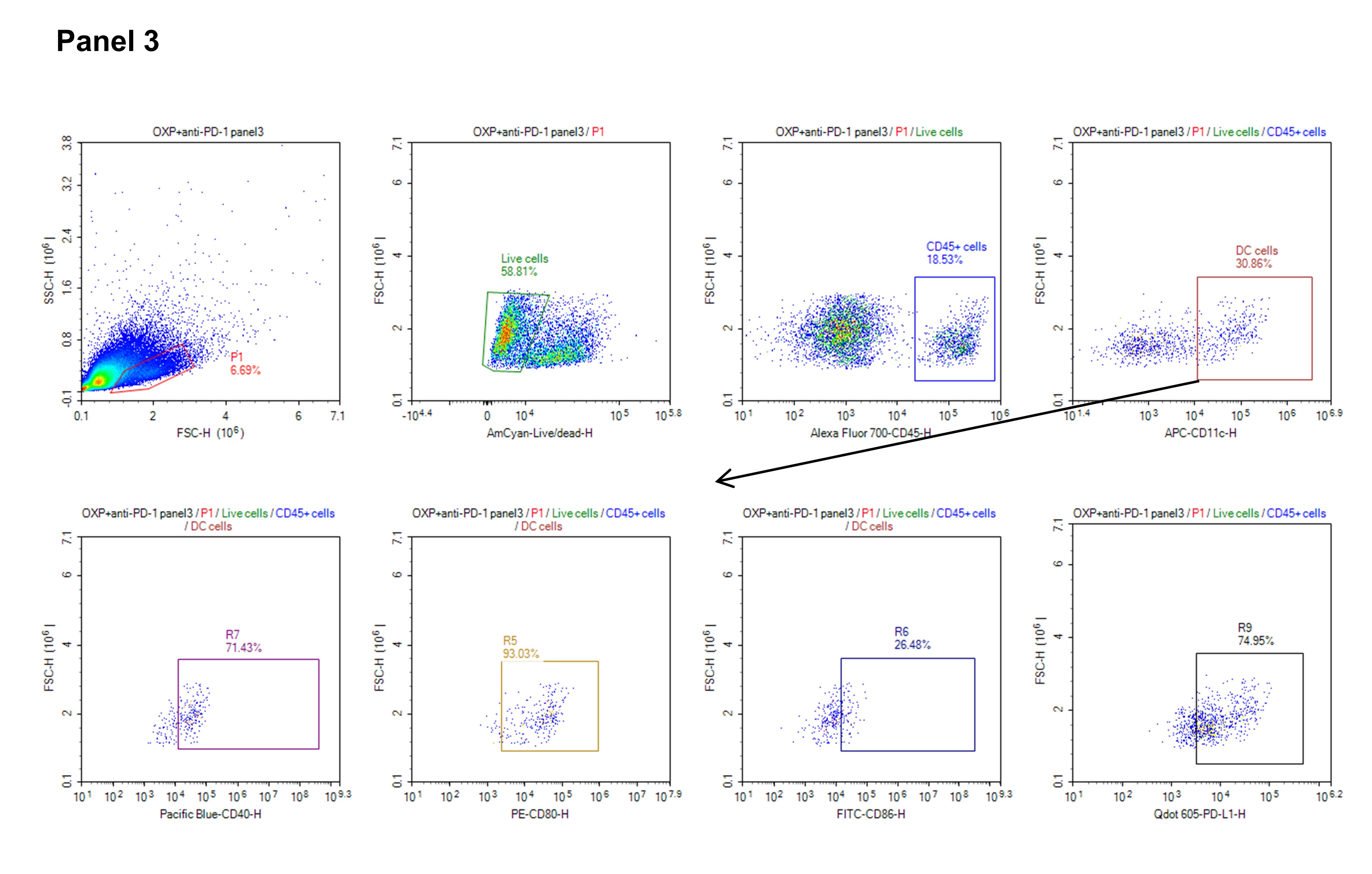

Supplement: Supplementary file 3 [file Image_3.jpeg]
